# A Phase IIIb, Multicentre, Randomised, Parallel-Group, Placebo-Controlled, Double-Blind Study to Investigate the Efficacy and Safety of OROS Hydromorphone in Subjects with Moderate-to-Severe Chronic Pain Induced by Osteoarthritis of the Hip or the Knee

**DOI:** 10.1155/2011/239501

**Published:** 2011-06-22

**Authors:** Jozef Vojtaššák, Jozef Vojtaššák, Adam Jacobs, Leonie Rynn, Sandra Waechter, Ute Richarz

**Affiliations:** ^1^Orthos Paidion, 83263 Bratislava, Slovakia; ^2^Dianthus Medical Limited, London SW19 2RL, UK; ^3^EMEA Medical Affairs, Analgesia, Janssen-Cilag, Quarryvale, Co Dublin, Ireland; ^4^Medical Affairs, Janssen-Cilag Europe, 6341 Baar, Switzerland; ^5^Global Medical Affairs, GMAL Mature Products, Johnson & Johnson Pharmaceutical Services, LLC, 6341 Baar, Switzerland

## Abstract

*Background*. Opioid analgesics are included in treatment guidelines for the symptomatic management of osteoarthritis (OA). Starting with a low dose of opioid and slowly titrating to a higher dose may help avoid intolerable side effects. *Methods*. Subjects aged ≥40 years, with moderate to severe pain induced by OA of the hip or knee not adequately controlled by previous non-steroidal anti-inflammatory drugs (NSAIDs) or paracetamol treatment, were enrolled. Subjects received OROS hydromorphone 4 mg or placebo once-daily. The dose was titrated every 3-4 days in case of unsatisfactory pain control during the 4-week titration phase. A 12 week maintenance phase followed. The primary efficacy endpoint was the change in “pain on average” measured on the Brief Pain Inventory (BPI) scale from baseline to the end of the maintenance phase. *Results*. 139 subjects received OROS hydromorphone and 149 subjects received placebo. All efficacy endpoints showed similar improvements from baseline to end of study in the 2 groups. The safety results were consistent with the safety profile of OROS hydromorphone. *Conclusion*.The study did not meet the primary endpoint; although many subjects' pain was not adequately controlled at inclusion, their pain may have improved with continued paracetamol or NSAID treatment.

## 1. Introduction

Providing relief from chronic pain is a major worldwide public health issue [1]. A systematic review of 4 studies that used the International Association for the Study of Pain (IASP) definition for chronic pain found that the prevalence of pain >3 months duration ranged from 11.5% to 55.2%; 3 studies involved the general population, and 1 study assessed patients in primary care [2]. A study of the general population in 15 European countries and Israel estimated that the point prevalence of chronic nonmalignant pain ranged from 12% to 30%; 40% of patients with chronic pain were not satisfied with the treatment they received [3]. Nearly 50% of individuals with chronic severe pain do not have their pain under control [4]. 

Osteoarthritis (OA) is the most common form of joint disease. The prevalence of OA increases with age; more than 75% of subjects over 70 years of age have radiologically detectable OA changes [5]. Clinical symptoms such as pain, joint stiffness, and joint deformities are associated with OA ([6–8]); OA leads to considerable restriction of mobility, which can progress to disability. OA was recently estimated to affect nearly 27 million adults in the United States [9]. There are currently no cures for OA, and treatment is aimed at managing the associated symptoms; treatments include reducing joint pain and stiffness, maintaining and improving joint mobility, reducing physical disability and handicap, improving health-related quality of life (HRQoL), limiting the progression of joint damage, and educating patients about the nature of the disorder and its management [10]. Therefore, OA is a major sociomedical problem with high prevalence and significant associated costs.

Opioid therapy is increasingly being used for the treatment of chronic noncancer pain although long-term opioid use remains controversial owing to concerns about long-term effectiveness and safety, particularly the risk of developing tolerance, dependence, or abuse ([11–17]). Common opioid side effects include nausea, vomiting, constipation, itching, and sedation; with the exception of constipation, these generally diminish over time [13]. Opioid side effects should be anticipated and treated as appropriate [18]. 

The use of opioid analgesics is included in treatment guidelines for the symptomatic management of OA of the hip or knee; weak opioids may be considered for the treatment of refractory pain where other pharmacological agents have been ineffective, whilst strong opioids should only be used for managing severe chronic pain in exceptional circumstances [10]. The effects on pain and function and the safety of oral or transdermal opioids compared with placebo or no intervention in patients with OA of the hip or knee was the subject of a recent Cochrane review [19]. Overall, opioids were more effective than control interventions in terms of pain relief and improvement of function, irrespective of the type or analgesic potency of the opioid. However, patients receiving opioids more frequently experienced adverse events (AEs) compared with patients receiving control interventions.

Guidelines on the use of opioids for chronic pain have recommended the use of controlled release formulations and scheduled regimens ([20–22]). Controlled-release opioid formulations aim to maintain sustained opioid plasma concentrations with minimal fluctuations between doses, offering sustained pain relief over 24 h with manageable side effects [23]. Starting with a low dose of opioid and slowly titrating to a higher dose may be beneficial for avoiding intolerable side effects. There is some limited evidence that this approach may be advantageous when starting treatment with oxycodone, oxymorphone, or hydromorphone ([24–27]).

OROS hydromorphone (Janssen Pharmaceutica NV, Beerse, Belgium) is a unique, once-daily opioid formulation that uses the patented OROS Push-Pull technology (ALZA, Vacaville, Calif, USA) to deliver hydromorphone with continuous, monophasic drug release over 24 hours, with the aim of providing consistent, longlasting analgesia ([28, 29]). In Europe, OROS hydromorphone is indicated for the treatment of moderate-to-severe chronic pain that needs continuous treatment and is currently available in 5 strengths: 4 mg, 8 mg, 16 mg, 32 mg, and 64 mg [28]. Not all dosage strengths may be available in all countries.

Here we report the results of the hydromorphone for OA pain (HOP) trial. In this placebo-controlled trial, subjects who were randomised to treatment with OROS hydromorphone hydrochloride were started on the lowest dose of 4 mg once daily; their dose was titrated every 3-4 days in the event of unsatisfactory pain control during the 4-week titration phase. The rationale for using a lower starting dose of 4 mg is that it may induce tolerance to the adverse effects of the opioid and therefore may reduce the number of AEs and consequently the dropout rate. The primary objective was to compare the analgesic effect of flexibly titrated OROS hydromorphone hydrochloride and placebo in subjects with moderate-to-severe pain induced by OA of the hip or knee that had not been adequately controlled by previous treatment with non-steroidal anti-inflammatory drugs (NSAIDs) or paracetamol measured by “pain on average” on the brief pain inventory (BPI) scale. Secondary objectives included assessing the dropout rate due to AEs, the effect of treatment on subjects' functionality using the total score of the Western Ontario and McMaster Universities (WOMAC) OA index, the effect of treatment on pain using the pain subscales of the WOMAC OA index and the HRQoL instrument short form (SF)-36 and pain-related items measured on the BPI scale, and the overall safety and tolerability of the drug. Secondary objectives analysed on an exploratory basis included assessing the effect of treatment on subjects' HRQoL using all other subscales, except pain, of the instrument SF-36, the effect of treatment on subjects' functional impairment and stiffness using these subscales of the WOMAC OA index, the effect of treatment on subjects' quality of sleep using a medical outcome study (MOS) sleep subscale score, and the dropout rate due to inefficacy.

## 2. Subjects and Methods

The study protocol and amendments were reviewed by the appropriate Independent Ethics Committee (IEC) or Institutional Review Board (IRB) for each study centre.

The study was conducted in accordance with the ethical principles that have their origin in the Declaration of Helsinki (amended Edinburgh 2000, with notes of clarification Washington 2002 and Tokyo, 2004) and that are consistent with good clinical practices (GCP) and applicable regulatory requirements.

Subjects gave written informed consent before entering the study.

### 2.1. Study Design, Population, and Treatments

This phase IIIb, multicentre, randomised, parallel-group, placebo-controlled, double-blind study was carried out at 18 sites in four European countries (Czech Republic, Romania, Slovakia, and UK). The study included male and female subjects aged ≥40 years, with moderate-to-severe pain induced by OA (as defined by the American College of Rheumatology) of the hip or knee. Moderate-to-severe OA pain was defined as a mean weekly score of ≥5 on a scale of 0–10 for “pain on average” on the BPI scale, which was calculated as a mean of the pain assessments collected at screening visit (week −1), telephone call (week –0.5), and baseline visit (week 0). Subjects must have suffered from chronic OA pain in the target joint for more than 3 months, and their pain must not have been adequately controlled with daily analgesic (NSAIDs or paracetamol) treatment for the month before beginning the study. Subjects were excluded from the study for any of the following reasons: regular treatment with an opioid in the 4 weeks before the screening visit—infrequent use of tramadol, codeine, tilidine, or dihydrocodeine for no more than 10 days in the 4 weeks before the screening visit was acceptable, but subjects were to stop any use of weak opioids at the screening visit, another type of continuous pain that stood out in comparison with OA pain such as fibromyalgia, cervical radiculopathy, or chronic low back pain, any of the following 6 months before entering study: major trauma to target joints, infection in target joints, radiologically apparent avascular necrosis in target joints, hyaluronan injections in the target joints, arthrodesis in the year or arthroscopy in the 2 months before entering study, planned treatment that could have altered the degree of pain within the study period, subjects who were being treated with buprenorphine, nalbuphine, or pentazocine; corticosteroid injections in the 3 months before the start of the study.

At the screening visit (week –1), subjects taking weak opioids discontinued their medication; subjects taking NSAIDs or paracetamol remained on a stable dose. Subjects were randomised to receive either oral OROS hydromorphone hydrochloride 4 mg once daily or matched placebo; placebo tablets were of identical appearance to the OROS hydromorphone hydrochloride tablets, that is matched for colour and size. Treatment comprised a 4-week titration phase and a 12-week maintenance phase. In the event of unsatisfactory pain control, subjects had their dose titrated 3-4 days after randomisation until week 4 of the study with intervals of at least 3-4 days between dose increments. Possible doses were 4 mg, 8 mg, 12 mg, 16 mg, 24 mg, and a maximum daily dose of 32 mg. There followed a 12-week maintenance phase on as stable a dose as possible. If a dose of 32 mg did not provide sufficient analgesia, subjects were withdrawn owing to lack of efficacy and had their dose tapered off by reducing their dose in specified increments every 2 days. At the end of the double-blind treatment phase, subjects had their dose tapered off to allow safe discontinuation of the study drug. This tapering off phase also applied if subjects prematurely discontinued. Paracetamol was allowed as rescue medication, provided that a subject did not exceed the total permitted daily dose (4 g per day until day 8 and then 2 g per day for the remainder of the study). 

Subjects returned to the clinic at 1, 2, 3, 4, 8, 12, and 16 weeks for scheduled assessments and were contacted by telephone call between visits. All items of the BPI were used to assess pain at screening, visit 1 (week 0), visit 5 (week 4), visit 6 (week 8), visit 7 (week 12), and visit 8 (week 16). Bodily pain, functional impairment, and stiffness were assessed using the WOMAC OA index at visit 1 (week 0), visit 5 (week 4), visit 6 (week 8), visit 7 (week 12), and visit 8 (week 16). HRQoL was assessed at visit 1 (week 0), visit 5 (week 4), visit 6 (week 8), visit 7 (week 12), and visit 8 (week 16) using the SF-36 HRQoL questionnaire. Quality of sleep was assessed at visit 1 (week 0) and visit 8 (week 16) using a MOS sleep subscale. AEs were monitored throughout the study. 

Subjects were randomly assigned to receive daily doses of OROS hydromorphone hydrochloride or placebo in a 1 : 1 ratio based on a computer-generated randomisation schedule prepared by an independent statistician (AJ). The randomisation was balanced by using randomly permuted blocks of subjects, with a block size of 4 and was stratified by OA of the hip or knee; study sites received blocks of study medication. There were two separate randomisation lists; one corresponded to OA of the hip and the other to OA of the knee. Each site received treatment kits for both strata (hip and knee), and the investigators were instructed to select the next available (consecutive) number for each of the strata. If subjects had OA of both the hip and knee, they were stratified according to whichever target joint was predominantly painful. Based on these randomisation lists, the study drug was packaged and labelled for the group of subjects according to the target joint. Subject numbers were preprinted on the study drug labels (distinctive identification numbers for hip and knee) and assigned as subjects qualified for the study. The medication kits were labelled with a 2-part tear-off label bearing information that met applicable regulatory requirements. One part of the tear-off label was attached to the subject's drug-dispensing log when the drug was dispensed. The investigator and the subject were blinded to treatment allocation.

### 2.2. Statistical Methods

#### 2.2.1. Sample Size Calculation

A difference of 1 point in “pain on average” on the BPI scale was considered to be a clinically important difference [30]. Based on the results of a previous study with OROS hydromorphone hydrochloride in subjects with OA [31], assuming that three baseline measures and seven post-baseline measures were collected, and calculating the sample size using the “sampsi” command in Stata version 8.2 (StataCorp, College Station, Texas) for repeated measures, 81 subjects were required per group to detect a difference of 1 point in the BPI measure with 90% power at a significance level of 5%. To allow for a dropout rate of approximately 40%, the study planned to recruit 135 subjects per group (i.e., 270 in total).

A reassessment of the sample size was done, without breaking the blind, when 50% of subjects were randomised and approximately 40% of subjects had either completed the study or dropped out. This analysis confirmed the assumptions of the sample size calculation.

#### 2.2.2. Analysis Populations

The safety population included all randomised subjects who received at least one dose of study drug. The intent-to-treat (ITT) population was used for efficacy analyses. The ITT population excluded subjects who had no post-baseline efficacy data and included subjects who discontinued early owing to lack of efficacy or other reasons. The per-protocol (PP) population was a subgroup of the ITT population that excluded subjects who discontinued early and all subjects with major protocol deviations. This population was used for supportive evidence for the efficacy analyses.

#### 2.2.3. Primary and Secondary Endpoints

The primary efficacy endpoint was assessed by recording the subjects' score in “pain on average” measured on the BPI scale at each study visit. Only data for the BPI collected at clinic visits was used. The primary efficacy endpoint was analyzed using a mixed-model regression analysis (mixed model for repeated measures (MMRM)), which took into account the correlation among repeated measures within individual subjects and allowed subjects with incomplete data (as a result of early dropout) to contribute their existing data to the analysis. The main model to test for the primary efficacy variable consisted of “pain on average” measured on the BPI scale as repeated measurement variable, treatment groups, time on study represented by the visit number, study joint, and baseline “pain on average” value (the latest pre-study treatment assessment) as fixed effects. The random effect was the subject number.

Secondary efficacy endpoints were the total score of the WOMAC OA index (visits 1, 5, 6, 7, and 8), pain as assessed using the pain subscales of the WOMAC OA index (visits 1, 5, 6, 7, and 8), pain as assessed using the pain subscales of the HRQoL instrument SF-36 (visits 1, 5, 6, 7, and 8), pain as assessed by “pain at its worst in the last 24 hours”, “pain at its least in the last 24 hours”, and “pain right now” measured on the BPI scale (visits 1, 5, 6, 7, and 8), all subscales except pain of the HRQoL instrument SF-36 (visits 1, 5, 6, 7, and 8), the functional impairment and stiffness subscales of the WOMAC OA index (visits 1, 5, 6, 7, and 8), the MOS sleep subscale (visits 1 and 8), the dropout rate due to AEs, and the dropout rate due to inefficacy. Safety assessment was based on reported AEs, dropouts due to AEs, vital sign measurements, and physical examinations. Secondary endpoints and safety results were summarised descriptively. AEs were coded using MedDRA dictionary version 11.0 (22 May 2008), and analyses were done using SAS Version 9.1.3.

#### 2.2.4. Post Hoc Analyses

Post hoc analyses were done to try to understand why the primary endpoint was not reached in this study. These included concomitant medication analyses to investigate, in an exploratory sense, the extent to which the primary objective of the study was compromised by use of concomitant analgesic medication, specifically paracetamol or NSAIDs. Analyses were done using Stata Version 9.2.

## 3. Results

### 3.1. Subjects Studied

The first subject attended the first study visit on 05 October 2007, and the last subject completed the last study visit on 24 November 2008.

**Figure 1:**
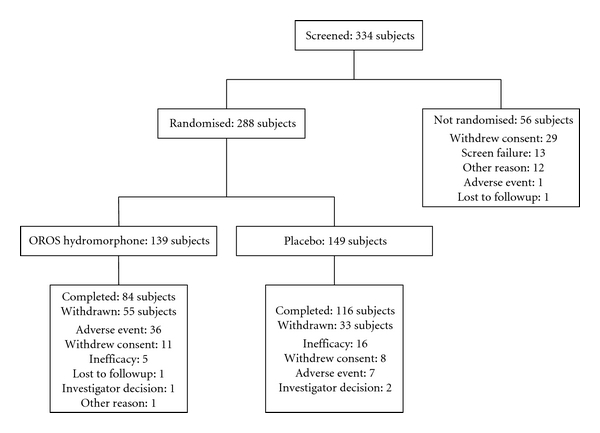
Flow of participants through the study.

344 subjects were screened and 288 were randomised: 139 subjects to the OROS hydromorphone group and 149 subjects to the placebo group. The maintenance phase was completed by 84 subjects in the OROS hydromorphone group and 116 subjects in the placebo group. In both groups, AE, inefficacy, and withdrawal of consent were the most common reasons for discontinuation of study treatment. Flow of subjects through the study is summarised in Figure 1.

The mean (standard deviation [SD]) age of subjects was 65 (±10.2) years. There were more subjects with OA of the knee (74%) than OA of the hip (26%). The baseline BPI, WOMAC OA index, HRQoL, and MOS sleep subscale scores results were similar for the OROS hydromorphone and placebo groups. Demography and baseline characteristics are summarised in Table 1.

The median (range) daily dose of study medication during the study, calculated from total dose used and the number of days on treatment, was 12.2 (3–28) mg and 20.1 (4–29) mg in the OROS hydromorphone and placebo groups, respectively.

### 3.2. Efficacy

**Figure 2:**
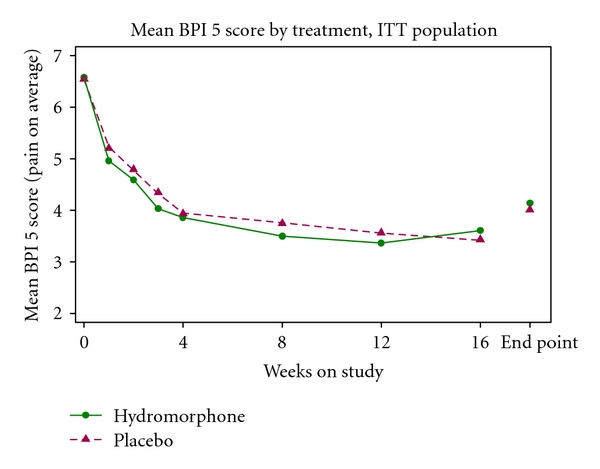
Mean change from baseline in BPI item 5 (pain on average) score by time (ITT population). Endpoint includes final assessments for subjects who discontinued before the end of the study.

The study did not meet the primary objective of showing superiority of OROS hydromorphone compared with placebo in its analgesic effect in subjects with moderate-to-severe pain induced by OA of the hip or knee, as measured by mean change from baseline in “pain on average” measured on the BPI scale. The primary efficacy analysis did not show superiority of OROS hydromorphone compared with placebo (difference −0.2365, 95% confidence interval −0.5357 to 0.0627; *P* = .1212). The factors “time on study” and baseline “pain on average” score measured on the BPI scale had a highly significant effect (*P* < .001) on the BPI scale “pain on average” score, which means baseline pain severity and time treated with the study drug had an influence on pain on average at endpoint; study joint (hip or knee) had no significant impact on the BPI scale “pain on average” score. Mean change from baseline in “pain on average” measured on the BPI scale score by time is shown in Figure 2, and mean change from baseline to the end of the maintenance phase is shown in see Table 2.

All secondary measures improved significantly in both groups from baseline to the end of the study. No statistically significant difference was found between treatment groups for any of the secondary endpoints looked at (see Table 2).

More subjects dropped out owing to an AE in the OROS hydromorphone group (25.9%; 36/139) than the placebo group (4.7%; 7/149); many of the AEs leading to discontinuation in the OROS hydromorphone group were typical opioid-related side effects, most commonly nausea and constipation. Subjects in the placebo group (10.7%; 16/149) more often dropped out owing to inefficacy compared with subjects in the OROS hydromorphone group (3.6%; 5/139).

### 3.3. Safety and Tolerability

The majority of TEAEs reported during the study were of mild or moderate severity: 208/232 TEAEs in the OROS hydromorphone group and 103/109 TEAEs in the placebo group. The frequency of mild TEAEs was comparable between treatment groups, 35% versus 30% of subjects in the OROS hydromorphone and placebo group, respectively. Higher proportions of subjects reported moderate and severe TEAEs in the OROS hydromorphone group (33% moderate and 14% severe) compared with placebo (12% moderate and 4% severe).

TEAEs that were possibly, probably, or very likely related to study drug were reported by 78% of subjects in the OROS hydromorphone group and 37% of subjects in the placebo group. Drug-related TEAEs were most frequently reported in the gastrointestinal disorders and nervous system disorders body systems in both groups; the frequency of these drug-related TEAEs was higher in the OROS hydromorphone group compared with the placebo group. Concomitant medications for the treatment of TEAEs were given to 48 (35%) subjects in the OROS hydromorphone group and 14 (9%) subjects in the placebo group. More subjects in the OROS hydromorphone group than the placebo group required treatment for drug-related constipation, nausea, and vomiting. Laxatives were used by 19.4% of subjects in the OROS hydromorphone group compared with 2.0% of subjects in the placebo group, and antiemetics were taken by 10.8% of subjects in the OROS hydromorphone group compared with 1.3% of subjects in the placebo group. 

One death (not considered related to study) occurred in the placebo group, no deaths occurred in the OROS hydromorphone group. There were 19 serious TEAEs reported during the study; 10 events occurred in 4 (2.9%) subjects in the OROS hydromorphone group, and 9 events occurred in 7 (4.7%) subjects in the placebo group.

### 3.4. Post Hoc Analyses

Post hoc analyses were done to try to determine the extent to which the primary objective of the study was compromised by use of concomitant paracetamol or NSAIDs. 

During the study, almost 100% of subjects received either NSAIDs or paracetamol either as concomitant medication or as rescue medication (Table 3).

**Figure 3:**
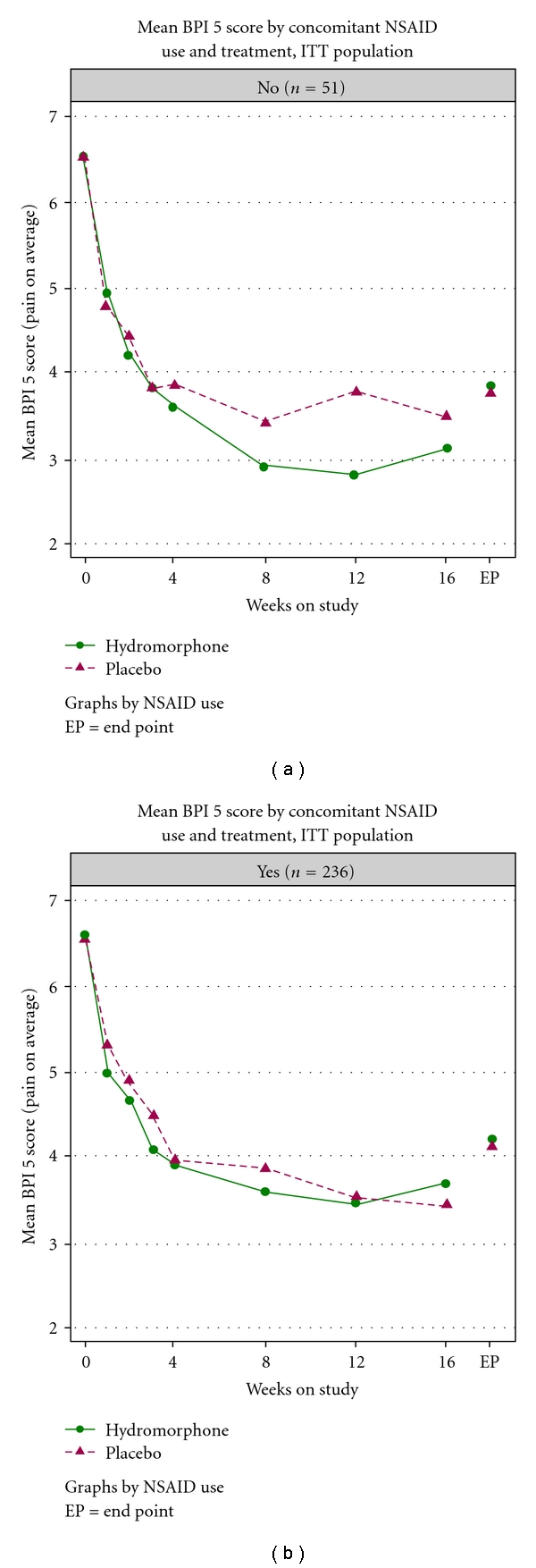
Mean BPI score item 5 (pain on average) by concomitant NSAID use and treatment (ITT population).

Post-baseline “pain on average” scores measured on the BPI scale were lower throughout the study for OROS hydromorphone compared with placebo in subjects not taking concomitant NSAIDs (*N* = 51; Figure 3). Scores were similar between groups in subjects who did take concomitant NSAIDs (*N* = 236). 

Post-baseline “pain on average” scores measured on the BPI scale were lower throughout the study for OROS hydromorphone compared with placebo in subjects taking paracetamol (*N* = 49). Scores were similar between groups in subjects who did not take concomitant paracetamol (*N* = 238).

## 4. Discussion and Conclusions

This randomised, double-blind, placebo-controlled, parallel-group trial compared the analgesic effect of flexibly titrated OROS hydromorphone hydrochloride and placebo in subjects with moderate to severe pain induced by OA of the hip or knee not adequately controlled by previous treatment with NSAIDs or paracetamol. The study included a high number of subjects with OA of the knee; more subjects with OA of the knee than with OA of the hip were available for enrolment into the study, possibly because total hip replacement is performed more frequently than total knee replacement.

The study did not meet the primary objective of showing superiority of OROS hydromorphone compared with placebo in its analgesic effect induced in subjects with moderate-to-severe OA of the hip or knee. The mean change from baseline in “pain on average” score on the BPI scale was similar in both groups. All secondary efficacy measures also improved significantly in both groups from baseline to the end of the study; these included total score of the WOMAC OA index, the pain subscale score of the WOMAC OA index, the pain subscale score of the SF-36 and “pain at its worst in the last 24 hours,” “pain at its least in the last 24 hours,” and “pain right now” on the BPI scale. The response observed in the placebo group may be explained by the placebo response commonly observed in pain studies ([32, 33]). However, the response in the OROS hydromorphone group was no better than that observed for the placebo group; these findings may be explained by a combination of the inclusion criteria and the use of concomitant medications.

Subjects were using non-opioid analgesics and NSAIDs before entering the study; based on expert scientific advice and ethical considerations, concomitant and rescue medication were permitted in the study design. Although subjects' pain was not adequately controlled with the concomitant analgesics at inclusion, subjects in the placebo group had a good response without side effects, whereas subjects in the OROS hydromorphone group got a good response with side effects. The strong placebo effect observed suggests that subjects may not have needed opioid treatment and their pain score (and other assessment results) would have improved with continued paracetamol and/or NSAID treatment [34]. Indeed, almost 100% of all subjects were treated with NSAIDs and/or paracetamol as concomitant medication. Post hoc concomitant medication analyses showed that in a subgroup of subjects taking NSAIDs, pain scores were similar with OROS hydromorphone and placebo treatment. In a subgroup of subjects not taking NSAIDs, pain scores were lower with OROS hydromorphone compared with placebo treatment. In a subgroup of subjects using concomitant paracetamol, pain scores appeared to be lower with OROS hydromorphone compared with placebo treatment. A previous study found that once-daily OROS hydromorphone was associated with effective pain relief and improved functionality in patients with chronic, moderate-to-severe OA of the knee or hip [27]. 

Subject selection may also have contributed to the outcome of this study. The subject selection criteria used may not have been stringent enough to ensure that only subjects with truly chronic pain were included; the mean “pain on average” score measured on the BPI scale was calculated from three assessments made during a one-week period from the screening visit to the baseline visit. Therefore, although subjects had sufficient pain severity to meet the inclusion criteria in the week immediately before the study, they may not have been experiencing pain from OA of that severity for a long period of time. It is, therefore, possible that some patients were experiencing a temporary exacerbation of pain that would have subsided with or without treatment. Comparison of the baseline WOMAC OA scores between the HOP trial and two previous placebo-controlled opioid studies suggest that the subjects in the HOP trial may have been less affected by their underlying illness compared with subjects in the other trials. In a randomised, placebo-controlled trial that recruited patients with OA requiring joint replacement and with moderate-to-severe pain that had been adequately controlled by weak opioids, transdermal fentanyl provided significantly better pain relief compared with placebo in patients even though previously prescribed NSAIDs and simple analgesics were continued [35]. In a study that recruited patients with chronic OA of the knee or hip who had moderate-to-severe mean daily pain intensity despite chronic use of stable doses of NSAIDs or other non-steroidal, non-opioid therapies, once-daily OROS hydromorphone was associated with effective pain relief and improved functionality [27]. Therefore, patients with more severe chronic pain may benefit from treatment with opioids.

During the study, drug-related TEAEs were reported by almost twice as many subjects in the OROS hydromorphone group compared with the placebo group. In both groups, the most frequently reported drug-related TEAEs were gastrointestinal and nervous system disorders; these events are commonly associated with opioids. No changes were observed in the well-documented safety profile of hydromorphone.

There is some limited evidence that starting with a low dose of opioid and slowly titrating to a higher dose may be advantageous for avoiding intolerable side effects when starting treatment with oxycodone, oxymorphone, or hydromorphone ([24–27]). The efficacy and tolerability of once-daily OROS hydromorphone and twice-daily ER oxycodone were compared in a 6-week, randomised, open-label, parallel-group study in patients with chronic, moderate-to-severe OA pain [27]. Patients received OROS hydromorphone initiated at a dose of 8 mg once daily (*n* = 71) versus ER oxycodone initiated at a dose of 10 mg twice daily (*n* = 67). The study consisted of a 14-day dose-titration and stabilisation period and a 28-day maintenance period. The knee was the affected joint in 79.8% of participants. Comparable levels of pain relief reductions in pain severity were observed. Some improvements were evident early in the study, when patients were receiving lower doses of analgesic. Dropouts due to AEs were similar between treatment groups; 35.2% in the hydromorphone group and 32.8% in the oxycodone group. 

In conclusion, the primary objective of showing superiority for OROS hydromorphone hydrochloride compared with placebo in its analgesic effect in subjects with moderate-to-severe pain induced by OA of the hip or knee was not met in this study. Secondary efficacy endpoints were also similar between the two treatment groups. Possible reasons for failure of the study are that subjects were not experiencing chronic moderate-to-severe pain or that their pain was adequately treated with paracetamol and/or NSAIDs. No changes were observed in the well documented safety profile of hydromorphone.

Before selecting patients with chronic OA pain for treatment with opioids, we recommend that clinicians be satisfied that pain is truly chronic and does not respond to weaker analgesics. A report published by the European league against rheumatism (EULAR) OA task force notes that opioid analgesics, with or without paracetamol, are useful alternatives in patients in whom NSAIDs, including COX-2 selective inhibitors, are contraindicated, ineffective, and/or poorly tolerated [36]. 

## Figures and Tables

**Table 1:** Demographics and baseline assessments of subjects in the HOP trial (ITT population).

	OROS hydromorphone *N* = 138	Placebo *N* = 149
Demographics		
Age (years); median (range)	65.0 (43–85)	66.0 (40–87)
Sex; *n* (%)		
Male	32 (23)	48 (32)
Female	106 (77)	101 (68)
Race; *n* (%)		
Caucasian	138 (100)	149 (100)
Weight (kg); mean (SD)	84.4 (15.7)	82.0 (15.6)
Height (cm); mean (SD)	163.9 (9.6)	165.9 (8.6)
Body mass index (kg/m^2^); mean (SD)	31.5 (5.6)	29.7 (5.0)
Most affected joint; *n* (%)		
Left knee	51 (37)	50 (34)
Right knee	54 (39)	58 (39)
Left hip	10 (7)	19 (13)
Right hip	23 (17)	22 (15)

*Baseline assessments*		
BPI; mean (SD)		
Pain at its worst in the last 24 h	7.8 (1.23)^1^	7.8 (1.06)^4^
Pain at its least in the last 24 h	4.6 (1.57)^1^	4.5 (1.50)^4^
Pain on average	6.6 (1.04)^1^	6.5 (0.94)^5^
Pain right now	6.4 (1.49)^1^	6.2 (1.67)^6^
WOMAC OA Index; mean (SD)		
Pain subscale	11.8 (2.63)^2^	11.5 (2.71)^5^
Functional impairment subscale	41.2 (9.25)^1^	39.8 (9.46)^4^
Stiffness subscale	4.6 (1.28)^2^	4.3 (1.44)^4^
Total score	17.7 (3.40)^3^	16.9 (3.90)^5^
SF-36; mean (SD)		
Pain subscale	27.7 (10.84)^2^	27.8 (11.01)^6^
Physical functioning subscale	25.0 (12.57)^1^	27.8 (13.33)^4^
Social functioning subscale	52.1 (20.82)^1^	52.0 (21.28)^4^
Mental health subscale	58.5 (17.47)^1^	59.5 (18.32)^4^
Health transition subscale	44.0 (13.74)^1^	43.0 (14.86)^5^
MOS sleep subscale		
Index I score	38.6 (17.43)^1^	35.7 (17.14)
Index II score	39.9 (17.08)^1^	37.2 (16.89)

^1^137 subjects; ^2^136 subjects; ^3^135 subjects; ^4^148 subjects; ^5^147 subjects; ^6^146 subjects.  *N*, total number of subjects; SD, standard deviation; *n*, number of subjects; BPI, brief pain inventory; h, hours; WOMAC OA, Western Ontario, and McMaster Universities Osteoarthritis; MOS, medical outcome study.

**Table 2:** Primary and secondary efficacy variables in the HOP trial: mean (standard deviation) change from baseline to end of maintenance phase (ITT population).

	OROS hydromorphone *N* = 138	Placebo *N* = 149
BPI		
Pain on average	–2.4 (2.1)^1^	–2.6 (2.3)^5^
Pain at its worst in the last 24 h	–2.4 (2.5)^1^	–2.7 (2.6)^5^
Pain at its least in the last 24 h	–1.7 (2.2)^1^	–1.6 (2.6)^5^
Pain right now	–2.6 (2.6)^1^	–2.4 (2.7)^6^
WOMAC OA Index		
Pain subscale	–3.74 (4.49)^2^	–3.86 (4.52)^5^
Functional impairment subscale	–11.93 (13.17)^1^	–11.90 (14.35)^6^
Stiffness subscale	–1.37 (1.85)^2^	–1.22 (1.84)^6^
Total score	–5.36 (5.99)^3^	–5.16 (6.15)^5^
SF-36 pain subscale	17.50 (20.48)^4^	19.47 (23.50)^7^
HRQoL		
Physical functioning subscale	13.59 (19.71)^1^	14.72 (24.08)^6^
Social functioning subscale	7.29 (23.42)^1^	9.55 (24.11)^6^
MOS Sleep Subscale		
Index I score	–5.77 (17.45)^1^	–5.65 (14.30)^6^
Index II score	–6.20 (16.81)^1^	–6.98 (14.43)^6^

^1^132 subjects; ^2^131 subjects; ^3^130 subjects; ^4^129 subjects; ^5^143 subjects; ^6^144 subjects; ^7^142 subjects. BPI, brief pain inventory; h, hour; WOMAC OA, Western Ontario and McMaster Universities Osteoarthritis; HRQoL, health-related quality of life; MOS, medical outcome study.

**Table 3:** Concomitant analgesic medication use in the HOP trial (ITT population).

	Treatment		
	OROS hydromorphone	Placebo	Total
NSAID, *n* (%)			
No	21 (15.22)	30 (22.13)	51 (17.77)
Yes	117 (84.78)	119 (79.87)	236 (82.23)
Total	138 (100)	149 (100)	287 (100)

Paracetamol, *n* (%)			
No	114 (82.61)	124 (83.22)	238 (82.93)
Yes	24 (17.39)	25 (16.78)	49 (17.07)
Total	138 (100)	149 (100)	287 (100)

Any analgesic, *n* (%)			
No	5 (3.62)	11 (7.38)	16 (5.57)
Yes	133 (96.38)	138 (92.62)	271 (94.43)
Total	138 (100)	149 (100)	287 (100)

ITT, intent-to-treat; NSAID, non-steroidal anti-inflammatory drug.
